# The normal distribution is not normal in psychological data: Moving beyond parametric dogma

**DOI:** 10.1371/journal.pmen.0000403

**Published:** 2025-08-12

**Authors:** Heury Ferr

**Affiliations:** Institute of Biological Sciences, Federal University of Goiás, Samambaia Campus, Goiânia, Goias, Brazil; PLOS: Public Library of Science, UNITED KINGDOM OF GREAT BRITAIN AND NORTHERN IRELAND

## Abstract

Psychological research has traditionally relied on parametric statistical methods, largely due to the historical convenience of the normal distribution. However, empirical evidence shows that psychological and mental health data often violate normality assumptions, exhibiting skewness, kurtosis, ordinal scaling, and outliers. Common constructs such as stress, anxiety, and substance use frequently display zero-inflated or asymmetric distributions, making parametric methods inappropriate and potentially misleading. Violations of normality increase the risk of Type I and II errors, bias effect estimates, and undermine inferential validity. While non-parametric tests offer more robust alternatives, modern resampling techniques such as bootstrapping and Monte Carlo simulations provide greater flexibility and accuracy without relying on strict distributional assumptions. This paper illustrates common deviations from normality in psychological data and advocates for a paradigm shift toward assumption-light analytical strategies. Emphasizing data visualization, transparent reporting, and statistical education, we argue for broader adoption of flexible methods to ensure more valid, interpretable, and reproducible findings in psychological science.

## Introduction

Psychological and mental health research has long relied on parametric analyses such as t-tests, ANOVA, and regression, largely because the normal distribution offered analytical convenience and accessible inference tables before the digital era [1,2]. This practicality cemented normality as a default assumption—often accepted uncritically—even though most psychological data rarely conform to it [3,4]. Instead, measures of complex, readouts like stress, anxiety, or attitudes tend to be skewed, kurtotic, ordinal, and contain outliers [4–8]. Persistently ignoring these features risks invalid results and flawed interpretations^5^.

Traditional non-parametric tests were developed as simpler, though sometimes less powerful, alternatives when assumptions clearly failed [4]. Today, with widespread computing power, researchers can move beyond outdated defaults by adopting flexible methods like resampling and Monte Carlo simulations [9]. While still requiring basic assumptions such as independence, these approaches do not rely on normality or homogeneity of variances. They generate empirical sampling distributions through repeated data shuffling or re-sampling, estimating p-values from the proportion of simulated statistics as extreme as the observed one [10]. This enables more accurate inference tailored to the true characteristics of psychological data.

## Illustrative examples: when normality is unlikely

Psychological variables often display distinctive, non-normal distribution shapes, which challenges the assumptions underlying many traditional statistical methods [3,5,6]. For example, occupational stress among call center workers typically clusters toward the upper end of measurement scales, resulting in markedly right-skewed distributions. These workers face intense pressure, strict monitoring, and emotionally taxing interactions, making the assumption of symmetric variability unrealistic [7].

Another frequent case arises with anxiety and depression symptoms in the general population. The majority of individuals tend to report minimal or no symptoms, while a small subset experiences severe distress. This leads to zero-inflated and skewed distributions, often accompanied by floor effects that violate normality assumptions [8].

A similar pattern is observed in substance use behavior. In community samples, many participants report no or minimal use, whereas a smaller group exhibits heavy, frequent usage. This creates not only zero-inflation and skewness, but sometimes multimodal distributions that are poorly captured by Gaussian-based models [1,8].

Even self-reported measures of social desirability or personality traits present challenges. Due to response biases, individuals tend to overstate positive attributes, leading to negatively skewed distributions with scores clustered near the maximum of the scale [1,9]. These are not statistical irregularities to be dismissed, but rather inherent properties of the constructs and the way they are measured.

Together, these examples underscore that ceiling and floor effects, skewness, and the discrete nature of psychological scales are common features—often systematically embedded in the data. Acknowledging these distributional patterns is critical for selecting appropriate analytical strategies and avoiding misleading inferences.

## Problems with parametric approaches

Despite their popularity, parametric tests can lead to flawed results when assumptions are violated [5,10]. These distributional violations may increase the risk of Type I errors (false positives)—detecting effects that are not actually present—or Type II errors (false negatives)—failing to detect effects that do exist. As a result, estimates can become biased and observed effects may be either exaggerated or masked. When assumptions are not met, the probability statements based on standard parametric tests may not be valid, leading to potentially misleading interpretations of data [11,12]. Data transformations like log or square-root may not fix non-normality or can complicate interpretation. Large samples do not guarantee robustness, especially with severe skewness or discrete scales [5]. Relying on parametric tests inappropriately risks overconfidence and misinterpretation, which can have serious consequences in applied settings. For instance, McGrath and Meyer (2006) demonstrated that assuming normality in psychological assessments can lead to incorrect classification decisions in clinical contexts, potentially affecting diagnosis and treatment planning [13]. Similarly, Hoekstra et al. (2012) highlighted how misunderstanding statistical assumptions contributed to misreporting of p-values and flawed inferences in psychology research, undermining evidence used to inform clinical guidelines [14].

## Alternatives: non-parametric and resampling methods

Non-parametric tests—including Mann–Whitney U, Wilcoxon signed-rank, Spearman correlations, and Kruskal–Wallis—do not require normality and handle ordinal, skewed data well. Although they are a valuable alternative when parametric assumptions are violated, non-parametric tests generally have less statistical power compared to their parametric counterparts (e.g., t-tests, ANOVA, Pearson correlation) when the data meet parametric assumptions [15].

Statistical power refers to the probability that a test will correctly reject a false null hypothesis. Resampling approaches like bootstrapping — a method that repeatedly draws samples from the data to estimate variability without relying on strong distributional assumptions — offer a robust alternative to traditional parametric tests.. Together, these tools provide flexible, accessible ways to analyze non-normal data [16,17]. Paired with transparent visualization, they reflect data structure more honestly and increase inferential accuracy.

## Recommendations

Psychologists should routinely visualize and summarize their data, reporting skewness, kurtosis, and distribution shapes. When normality is questionable, researchers should prioritize non-parametric or resampling approaches and avoid forced transformations solely to satisfy parametric assumptions.

Statistical training must emphasize recognizing distributional violations and selecting flexible, assumption-light tools. Both students and faculty would benefit from more structured exposure to applied statistical reasoning and from consulting with statisticians as a routine part of the research process.

Furthermore, encouraging open data practices—such as sharing anonymized datasets as supplementary material—can allow independent verification, foster secondary analyses, and ultimately promote more rigorous and replicable psychological science.

## Final considerations

Assuming normal distribution in psychological data is frequently unjustified—a relic of statistical convention, not reality. Many psychological variables are intrinsically skewed, bounded, or categorical. Abandoning unjustified assumptions and adopting flexible analyses tailored to empirical data will enhance the validity, transparency, and rigor of psychological science and mental health.

## References

[1] David HA. Tables Related to the Normal Distribution. The American Statistician. 2005;59(4):309–11. doi: 10.1198/000313005x70911

[2] Kauermann G, Küchenhoff H, Heumann C. Bootstrapping. Springer Series in Statistics. Springer International Publishing. 2021. p. 197–229. doi: 10.1007/978-3-030-69827-0_8

[3] Counsell N, Cortina-Borja M, Lehtonen A, Stein A. Modelling psychiatric measures using Skew-Normal distributions. Eur Psychiatry. 2011;26(2):112–4. doi: 10.1016/j.eurpsy.2010.08.006 21036551 PMC3080602

[4] van den Oord EJCG, Pickles A, Waldman ID. Normal variation and abnormality: an empirical study of the liability distributions underlying depression and delinquency. J Child Psychol Psychiatry. 2003;44(2):180–92. doi: 10.1111/1469-7610.00112 12587855

[5] Blanca MJ, Arnau J, López-Montiel D, Bono R, Bendayan R. Skewness and Kurtosis in Real Data Samples. Methodology. 2013;9(2):78–84. doi: 10.1027/1614-2241/a000057

[6] Sawada T. Conditions of the Central-Limit Theorem Are Rarely Satisfied in Empirical Psychological Studies. Front Psychol. 2021;12:762418. doi: 10.3389/fpsyg.2021.762418 34858289 PMC8630578

[7] Yuan KH, Bentler PM. Normal theory based test statistics in structural equation modelling. Br J Math Stat Psychol. 1998;51(Pt 2):289–309. doi: 10.1111/j.2044-8317.1998.tb00682.x 9854947

[8] Counsell N, Cortina-Borja M, Lehtonen A, Stein A. Modelling psychiatric measures using Skew-Normal distributions. Eur Psychiatry. 2011;26(2):112–4. doi: 10.1016/j.eurpsy.2010.08.006 21036551 PMC3080602

[9] Lamberti R, Petetin Y, Desbouvries F, Septier F. Independent Resampling Sequential Monte Carlo Algorithms. IEEE Trans Signal Process. 2017;65(20):5318–33. doi: 10.1109/tsp.2017.2726971

[10] Boos DD, Zhang J. Monte Carlo Evaluation of Resampling-Based Hypothesis Tests. Journal of the American Statistical Association. 2000;95(450):486–92. doi: 10.1080/01621459.2000.10474226

[11] BONEAU CA. The effects of violations of assumptions underlying the test. Psychol Bull. 1960;57:49–64. doi: 10.1037/h0041412 13802482

[12] Glass GV, Peckham PD, Sanders JR. Consequences of Failure to Meet Assumptions Underlying the Fixed Effects Analyses of Variance and Covariance. Review of Educational Research. 1972;42(3):237–88. doi: 10.3102/00346543042003237

[13] McGrath RE, Meyer GJ. When effect sizes disagree: the case of r and d. Psychol Methods. 2006;11(4):386–401. doi: 10.1037/1082-989X.11.4.386 17154753

[14] Hoekstra R, Morey RD, Rouder JN, Wagenmakers E-J. Robust misinterpretation of confidence intervals. Psychon Bull Rev. 2014;21(5):1157–64. doi: 10.3758/s13423-013-0572-3 24420726

[15] Kitchen CMR. Nonparametric vs parametric tests of location in biomedical research. Am J Ophthalmol. 2009;147(4):571–2. doi: 10.1016/j.ajo.2008.06.031 19327444 PMC2743502

[16] Fieberg JR, Vitense K, Johnson DH. Resampling-based methods for biologists. PeerJ. 2020;8:e9089. doi: 10.7717/peerj.9089 32419987 PMC7211410

[17] Bland JM, Altman DG. Statistics Notes: Bootstrap resampling methods. BMJ. 2015;350:h2622. doi: 10.1136/bmj.h2622 26037412

